# Performance in youth track and field is associated with birth quartile. A register-based study among athletes in Norway from 10 years to senior level

**DOI:** 10.1371/journal.pone.0273472

**Published:** 2022-09-06

**Authors:** Hilde Gundersen, Anette Harris, Halvard Grendstad, Morten Kristoffersen, Atle Guttormsen, Terje Dalen, Cecilie Brekke Rygh

**Affiliations:** 1 Department of Sport, Food and Natural Sciences, Western Norway University of Applied Sciences, Bergen, Norway; 2 Department of Psychosocial Science, Faculty of Psychology, University of Bergen, Bergen, Norway; 3 Department of Physical Performance, Norwegian School of Sport Sciences, Oslo, Norway; 4 NMBU School of Economics and Business, Norwegian University of Life Sciences (NMBU), Ås, Norway; 5 Department of Physical Education and Sport Science, Faculty of Teacher Education and Arts, Nord University, Levanger, Norway; 6 Department of Health and Functioning, Western Norway University of Applied Sciences, Bergen, Norway; Nottingham Trent University, UNITED KINGDOM

## Abstract

**Introduction:**

Earlier studies have demonstrated that the oldest in a competition class are more likely to succeed than the youngest, a phenomenon called relative age effect (RAE). Track and field give us an opportunity to investigate the advantage of being born early in the year based upon actual performance, since objective criteria are the performance indicators. Hence, the aim of the present study was to investigate the occurrence of RAE in Norwegian track and field athletes in events where physical capacity is important for success.

**Methods:**

All individual season best results from the register of The Norwegian Athletics Federation (n = 28 999) obtained in all competition classes from the age of 10 years to senior in both sexes on 60m and 600m from 2011 to 2020 were downloaded. One-way ANOVA and LSD post hoc analyses were used to analyze performance differences according to birth quartiles between athletes. Further, odds ratios (OR) were used to calculate the odds of being among the top-100 for athletes for those born in the first quartile of the year compared to the last.

**Results:**

The RAE was present in several of the competition classes in sprint compared to middle-distance running, and in more male than female competition classes. Overall, the OR of being among the top-100 in one of the competition classes on 60m sprint when born in first quartile compared to last quartile was 2.88 [2.30–3.62] for males and 1.54 [1.26–1.89] for females.

**Conclusion:**

Being born early in the year in events with high demand for specific physical capacities is an advantage in both sexes in most of the youngest competition classes. In males, the advantage of being born early in the year lasted longer in sprint than in middle-distance running, indicating that puberty affects performance in sprint and middle-distance running differently.

## Introduction

Organized sport is one of the most popular forms of leisure-time activities worldwide, and at least one-third of children and adolescents are participating in one or more sports in most countries [1]. To provide equal opportunities and competition, children and youth usually compete in age classes based on their chronological age. However, previous studies have shown that children born early in their birth year are likely to perform better than children born later in the same age cohort, a phenomenon called relative age effect (RAE) [2]. The maturational hypothesis is perhaps the most common explanation for the RAE, i.e. that chronologically older children have a higher chance of being more physically mature, with subsequent anthropometric and physiological characteristics that aid performance [3–9].

It is reasonable to expect that the RAE would be more prominent for younger athletes because of the age differences being relatively larger. During puberty, the RAE is often found to be strongest in this period related to the large variation in physique and anthropometry due to age [10]. After puberty, RAE are often found to subside [11], however, secondary effects may maintain the persistence during senior carrier [12]. Early maturation and success may increase training motivation and thus the sport-specific skills and experience [11, 13]. Furthermore, those who perform at highest level in youth sports may be favoured by more competent coaches, more systematic training and better training facilities [14, 15]. Consequences of the RAE may include favouring the physically mature at the expense of those less mature, and in worst case athletes dropout from the sports because of late maturation [11, 16].

RAE is well documented in team sports [2, 17], but there is still less evidence regarding RAE in individual sports [17]. There are, however, some important differences between team sports and individual sports that may influence the occurrence of RAE. The selection that occurs in team sports are not seen to the same extent in individual sports. In individual sports, success is also related to individual performance and not confounded by inter-individual factors like team formations, tactics and positional roles [18]. Performances are further judged on objective data in most individual sports, rather than of a subjective evaluation of an individual’s contribution to a team performance. RAE-studies in team sports are most often investigating the advantage/disadvantage for the selected/non-selected players, and this selection are based upon subjective criteria. Track and field give us an opportunity to investigate the advantage of being born early in the year based upon actual performance, since objective criteria are the performance indicators.

There are few previous studies investigating RAEs in track and field, and as far as we know, only one study from the Scandinavian countries [19]. In addition, the majority of previous studies in track and field have focused on athletes who are 14 years (U15) or older who compete at international level [20–26]. The occurrence of RAE has further been investigated more in males than females [19, 20, 23, 27, 28]. Since the Norwegian Athletics Federation has registered seasonal best for each athlete in all events obtained in official competitions from the age of 10 years to senior the last decades, the register will provide as an unique opportunity to investigate RAE in track and filed from child to adult in both sexes. Since biological maturation is suggested as an explanation for the RAE, the aim of the present study was to investigate the occurrence of RAE in events where physical capacity is important for the result in all competition classes, in both sexes. We aimed to use two different approaches 1) to investigate performance differences between athletes born in different quartiles of the year in all competition classes in sprint (60m) and middle-distance running (600m) in both sexes, and 2) to calculate the OR of being among the top-100 athletes in each of the competition class for those born in first vs. last quartile of the year in all competition classes for both sexes.

## Methods

The Norwegian Athletics Federation has the last decades registered all results obtained in official competitions by Norwegian athletes. The register includes an overview of the best result of each athlete in each event in all competition classes, from 10 years to senior. For each result, competition class and competition date are registered. Birth year is registered for all athletes, and birth date for most of the athletes. The present study was conducted in accordance with the declaration of Helsinki and approved by the Norwegian centre for research data (NSD) (324455) and the Norwegian Athletics Association. Since the data are based on publicly available resources, no informed consent was obtained.

### Procedures

Results available on 60m and 600m in the database from 2011 to 2020 were downloaded on the 1st of January 2021. Only indoor results were included to avoid differences in wind and temperature conditions, and since 60m is the main sprint distance indoor in all competition classes. 60m is an international distance indoors. In Norway, youth athletes are organized within one-year age bands from the age of 10 until the age of 17 years, and in two-years age band for 18 and 19 years old (U20). Senior included results obtained from the age between 20 and 34 years, although athletes can compete in the senior class when 15 years old in Norway. Only the best result for each athlete in each competition classes were included in the analyses. Results obtained without electronic timing and athletes without registered birth date were excluded. Birth months was categorised in birth quartiles; Q1: January-March, Q2: April-June, Q3: July-September, Q4: October-December.

### Data analyses

Data are presented as mean with standard deviation (SD) or as frequencies. Visual inspection confirmed that all data were normally distributed. One-way ANOVA analyses were performed to analyse whether there were differences between results obtained from athletes born in different quartiles (Q1, Q2, Q3 and Q4) for each competition class and event, separated by sex. Post hoc analyses were performed with the least significant difference (LSD) test. Frequencies of the top 100 athletes born in each competition class and event for females and males were calculated. Crosstab analyses were performed to assess odds ratio (OR) and 95% confidence interval [CI] of being among the top 100 athletes (yes/no) for those born in first vs. fourth quartile of the year. IBM SPSS Statistics (version 27) was used for all statistical analyses and statistical significance was accepted at p < 0.05.

## Results

Totally, 28 999 results were registered along with birth date for 60m sprint and 600m middle distance running, 15 244 and 13 755 results for females and males respectively. For 60m sprint, a total of 21 419 results were registered (11 512 for females and 9907 for males), and for 600m middle distance running, a total of 7580 results were registered (3732 for females and 3848 for males).

### Result difference between athletes born in different quartiles of the year

**Fig 1:**
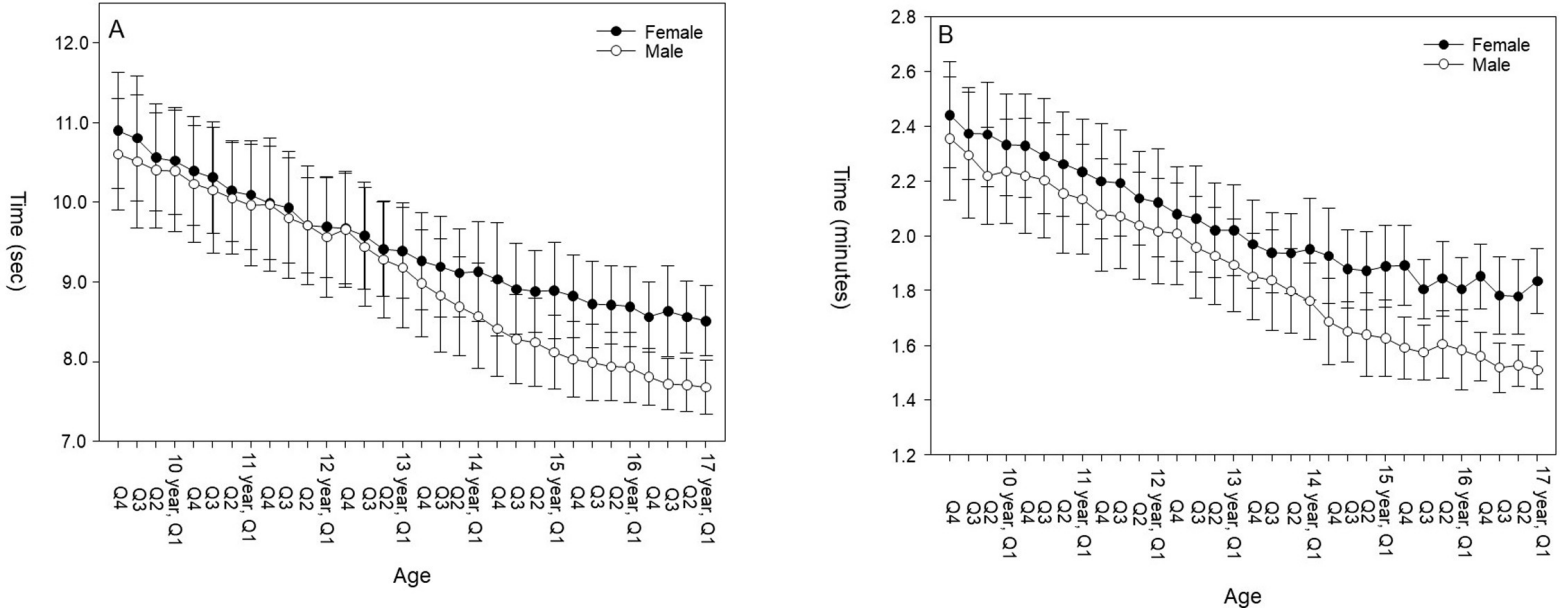
Mean of best indoor running times on 60-meter sprint (A) and 600-meter middle-distance running (B) for different age groups in male and female track and field athletes from 2011–2020. Q1: born in January-March, Q2: born April-June, Q3: born in July-September, Q4: born in October-December.

There were significant differences in results obtained by athletes born in different quartiles, especially among the youngest athletes. Being born in the first quartile was an advantage regarding performance compared to being born in the last quartile for both events in all competition classes from 11 to 13 years (See Fig 1 and Tables 1 and 2 for more specific information). In sprint, there were better results in first quartile compared to the last quartile in all completion classes until the age of 16 years in females (Table 1 and Fig 1), and in most of the competition classes until U20 in males (Table 2 and Fig 1). In middle-distance running (600m), there were better results in first quartile compared to the last quartile in all competition classes until the competition class 13 year in females (Table 1 and Fig 1), and until 14 years in males (Table 2 and Fig 1). In females, the largest main effect was seen in the competition class 13 years on 60m sprint performance and in the competition class 12 years on 600m middle distance running. There was no significant result difference between quartiles from the age of 17 years in any of the two events. In males, the largest main effect was seen in the competition class 13 years for both events. No significant result difference was found at senior level in sprint performance and from the age of 15 years on middle-distance running performance. For an overview of number of athletes born in each quartile see S1 and S2 Tables.

**Table 1:** Differences in performance on 60m sprint and 600m middle-distance running in females born in different birth quartiles (Q) for each competition class. Data are presented as mean ± standard deviation.

Competition classes (years)	Q1	Q2	Q3	Q4	Main effect	Post hoc analyses LSD
	January-March	April-June	July-August	Sept.-October		
**60m sprint** (sec)						
10 (n = 842)	10.52 ±0.67	10.56 ±0.67	10.80 ±0.78	10.90 ±0.73	F(3,838) = 13.45, p < .001	Q1>Q3, Q4, Q2>Q3, Q4
11 (n = 1367)	10.09 ±0.68	10.14 ±0.63	10.31 ±0.70	10.39 ±0.68	F(3,1363 = 14.64, p < .001	Q1>Q3, Q4, Q2>Q3, Q4
12 (n = 1795)	9.69 ±0.63	9.71 ±0.60	9.93 ±0.70	9.99 ±0.71	F(3,1791) = 22.38, p < .001	Q1>Q3, Q4, Q2>Q3, Q4
13 (n = 2184)	9.39 ±0.60	9.41 ±0.59	9.58 ±0.67	9.67 ±0.69	F(3,2180) = 23.68, p < .001	Q1>Q3, Q4, Q2>Q3, Q4
14 (n = 1828)	9.13 ±0.63	9.11 ±0.55	9.19 ±0.63	9.26 ±0.61	F(3,1824) = 5.19, p < .001	Q1>Q4, Q2>Q4
15 (n = 1300)	8.89 ±0.60	8.88 ±0.51	8.91 ±0.57	9.03 ±0.71	F(3,1296) = 4.02, p = .007	Q1>Q4, Q2>Q4
16 (n = 920)	8.69 ±.050	8.71 ±0.49	8.72 ±0.54	8.82 ±0.52	F(3,916) = 2.90, p = .034	Q1>Q4
17 (n = 604)	8.51 ±0.44	8.56 ± 0.45	8.63 ±0.57	8.56 ±0.44	F(3,600) = 1.55, p = .201	-
U20 (n = 508)	8.42 ±0.54	8.42 ±0.43	8.43 ±0.46	8.48 ±0.52	F(3,504) = 0.40, p = .752	-
Senior (n = 162)	8.09 ±0.47	8.18 ±0.45	8.23 ±0.61	8.22 ±0.54	F(3,158) = 0.64, p = .592	-
**600m middle distance running** (min)						
10 (n = 347)	2.19.87 ±11.15	2.22.17 ±11.40	2.22.31 ±9.99	2.26.24 ±11.58	F(3,343) = 3.95, p < .009	Q1>Q4, Q2>Q4, Q3>Q4
11 (n = 631)	2.13.96 ±11.60	2.15.64 ±11.39	2.17.37±12.56	2.19.68 ±11.36	F(3,627) = 6.17, p < .001	Q1>Q3, Q4, Q2>Q4
12 (n = 758)	2.07.23 ±11.79	2.08.19 ±10.32	2.11.49±11.55	2.11.87 ±12.58	F(3,754) = 7.66, p < .001	Q1>Q3, Q4, Q2>Q3, Q4
13 (n = 762)	2.01.19 ±10.01	2.01.16 ±10.31	2.03.69±11.64	2.04.71 ±10.44	F(3,758) = 5.12, p < .002	Q1>Q3, Q4, Q2>Q3, Q4
14 (n = 587)	1.57.02 ±11.23	1.56.11 ±8.73	1.56.22 ±8.73	1.58.12 ±9.65	F(3,583) = 1.07, p = .360	-
15 (n = 256)	1.53.27 ±8.97	1.52.31 ±8.59	1.52.71 ±8.54	1.55.56 ±10.42	F(3,252) = 1.28, p = .282	-
16 (n = 155)	1.48.27 ±6.99	1.50.61 ±8.19	1.48.25 ±6.47	1.53.48 ± 8.71	F(3,151) = 3.53, p = .016	Q1>Q4, Q3>Q4
17 (n = 120)	1.50.04 ±10.09	1.46.67 ±8.18	1.46.94 ±8.50	1.51.10 ±7.08	F(3,116) = 1.77, p = .158	-
U20 (n = 77)	1.43.71 ±6.86	1.43.81 ±6.04	1.42.75 ±7.17	1.44.03 ±6.67	F(3,73) = 0.13, p = .940	-
Senior (n = 39)	1.42.42 ±11.17	1.38.49 ±2.65	1.42.47 ±10.24	1.41.88 ±7.74	F(3,35) = 0.47, p = .706	-

U20: 18–19 years, Senior: 20–34 years. > indicate significant better performance for athletes born in quartiles at the left side of the sign compared to those born in quartiles at the right side.

**Table 2:** Differences in performance on 60m sprint and 600m middle-distance running for males born in different birth quartiles (Q) for each competition class. Data are presented as mean ± standard deviation (SD).

Competition classes (years)	Q1	Q2	Q3	Q4	Main effect	Post hoc analyses LSD
	January-March	April-June	July-August	Sept.-October		
**60m** (sec)						
10 (n = 710)	10.39 ±0.76	10.40 ±0.72	10.51 ±0.84	10.60 ±0.70	F(3,706) = 2.59, p = .052	-
11 (n = 1118)	9.96 ±0.76	10.05 ±0.70	10.15 ±0.79	10.23 ±0.73	F(3,1114) = 6.19, p < .001	Q1>Q3, Q4, Q2>Q4
12 (n = 1406)	9.56 ±0.75	9.71 ±0.75	9.80 ±0.76	9.97 ±0.83	F(3,1402 =) 16.31, p < .001	Q1>2Q, 3Q, 4Q, 2Q>4Q 3Q>4Q
13 (n = 1735)	9.18 ±0.75	9.28 ±0.73	9.44 ±0.74	9.66 ±0.73	F(3,1731) = 31.21, p < .001	Q1>Q2, Q3, Q4, Q2>Q3, Q4, Q3>Q4
14 (n = 1335)	8.57 ±0.66	8.69 ±0.62	8.83 ±0.71	8.98 ±0.67	F(3,1331) = 22.81, p < .001	Q1>Q2, Q3, Q4, Q2>Q3, Q4, Q3>Q4
15 (n = 1081)	8.12 ±0.46	8.24 ±0.55	8.28 ±0.56	8.41 ±0.60	F(3,1077) = 11.49, p < .001	Q1>Q2, Q3, Q4, Q2>Q4, Q3>Q4
16 (n = 880)	7.93 ±0.44	7.94 ±0.43	7.99 ±0.48	8.03 ±0.47	F(3,876) = 2.15, p = .092	-
17 (n = 645)	7.68 ±0.34	7.71 ±0.33	7.72 ±0.32	7.81 ±0.36	F(3,641) = 4.29, p = .005	Q1>Q4, Q2>Q4, Q3>Q4
U20 (n = 634)	7.55 ±0.35	7.58 ±0.38	7.64 ±0.44	7.67 ±0.37	F(3,630) = 3.25, p = .022	Q1>Q3, Q4, Q2>Q4
Senior (n = 363)	7.45 ±0.48	7.49 ±0.47	7.51 ±0.45	7.52 ±0.47	F(3,359) = 0.45, p = .718	-
**600m middle distance running** (min)						
10 (n = 363)	2.14.06 ±11.41	2.13.03 ±10.61	2.17.61 ±13.79	2.21.28 ±13.47	F(3,359) = 7.67, p < .001	Q1>Q3, Q4, Q2>Q3, Q4
11 (n = 628)	2.07.94 ±12.04	2.09.20 ±13.03	2.12.11 ±12.46	2.13.09 ±12.62	F(3,624) = 5.41, p < .001	Q1>Q3, Q4, Q2>Q3, Q4
12 (n = 710)	2.00.92 ±11.50	2.02.26 ±11.74	2.04.17 ±11.39	2.04.59 ±12.29	F(3,706) = 3.72, p < .011	Q1>Q3, Q4
13 (n = 808)	1.53.56 ±10.14	1.55.55 ±10.61	1.57.38 ±11.09	2.00.45 ±11.11	F(3,804) = 14.85, p < .001	Q1>Q3, Q4, Q2>Q4, Q3>Q4
14 (n = 559)	1.45.66 ±8.43	1.47.86 ±9.24	1.50.21 ±10.98	1.51.04 ±9.48	F(3,555) = 9.12, p < .001	Q1>Q2, Q3, Q4 Q2>Q3, Q4
15 (n = 250)	1.37.57 ±8.04	1.38.30 ±6.69	1.39.00 ±6.58	1.41.20 ±9.35	F(3,246) = 2.34, p = .074	-
16 (n = 193)	1.35.02 ± 8.71	1.36.22 ± 7.43	1.34.45 ±6.06	1.35.45 ±6.82	F(3,189) = 0.51, p = .675	-
17 (n = 129)	1.30.58 ±4.16	1.31.63 ±4.45	1.31.12 ±5.44	1.33.56 ±5.26	F(3,125) = 2.06, p = .110	-
U20 (n = 128)	1.27.15 ±4.15	1.28.84 ±5.96	1.28.05 ±4.59	1.28.98 ±4.05	F(3,124) = 1.04, p = .379	-
Senior (n = 80)	1.27.42 ±9.16	1.31.54 ±12.02	1.28.74 ±9.12	1.26.28 ±4.56	F(3,76) = 1.00, p = .398	-

U20: 18–19 years, Senior: 20–34 years> indicate significant better performance for athletes born in quartiles at the left side of the sign compared to those born in quartiles at the right side.

### OR of being among the top-100 athletes

**Fig 2:**
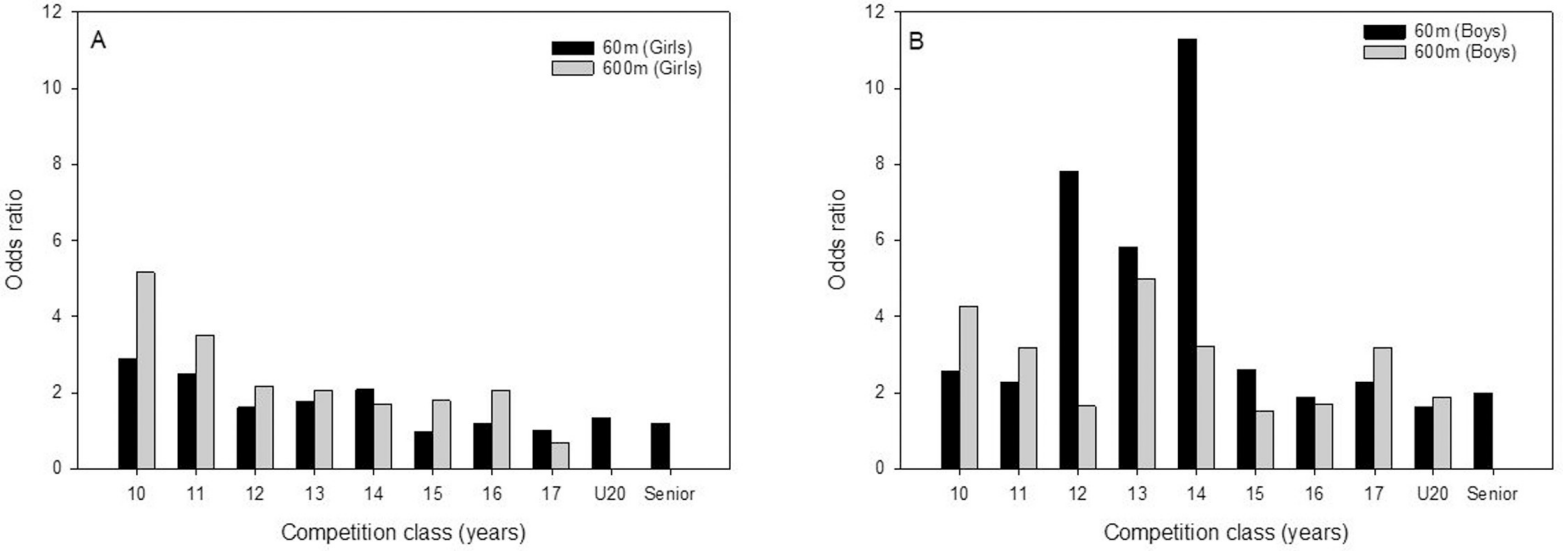
An overview of odds ratio (OR) of being among the top 100 athletes and born in Q1 compared to Q4 for girls (A) and boys (B).

There were higher ORs for being among the top-100 athletes when born in first quartile of the year compared to the last quartile. Overall, the OR of being among the top-100 in one of the competition classes on 60m sprint when born in first quartile compared to last quartile was 2.88 [2.30–3.62] for males and 1.54 [1.26–1.89] for females. Similar, overall OR of being among top-100 athletes on 600m middle-distance running was 2.05 [1.64–2.54] and 1.88 [1.48–2.38] for males and females, respectively. In females, highest ORs were seen in the competition class 10 years in both events, although significantly higher OR was seen until competition class 14 years on 60m and until 13 years on 600m. In males, highest ORs were seen in competition class 14 years on sprint, and in competition class 13 years on 600m. Significant higher OR was seen in most competition classes until senior level on 60m and until the competition class 14 years on 600m (see Table 3 and Fig 2 for more details).

**Table 3:** On overview over OR 95% [CI] of being among the top-100 athletes and born in Q1 compared to Q4.

	Females		Males	
Competition class (years)	60m sprint	600m	60m sprint	600m
10	2.89 [1.36–6.13]	5.15 [2.03–13.06]	2.54 [1.18–5.49]	4.26 [1.77–10.29]
11	2.49 [1.29–4.81]	3.50 [1.64–7.497]	2.26 [1.10–4.66]	3.17 [1.42–7.09]
12	1.60 [.85–3.01]	2.16 [1.11–4.179]	7.82 [3.08–19.85]	1.64 [.88–3.06]
13	1.76 [.87–3.59]	2.06 [1.03–4.149]	5.81 [2.62–12.87]	4.97 [2.37–10.43]
14	2.07[1.06–4.05]	1.69 [.83–3.432]	11.27 [3.49–36.45]	3.21 [1.52–6.75]
15	.96 [.53–1.74]	1.78 [.81–3.931]	2.59 [1.23–5.45]	1.50 [.72–3.14]
16	1.19 [.64–2.22]	2.04 [.77–5.393]	1.88 [.93–3.80]	1.68 [.74–3.82]
17	1.01 [.52–1.94]	.69 [.19–2.520]	2.28 [1.14–4.57]	3.18 [.92–11.03]
U20 (18–19)	1.34 [.70–2.59]	*	1.62 [.86–3.05]	1.87 [.55–6.32]
Senior (20–34)	1.19 [.49–3.03]	*	1.98 [1.00–3.91]	*

* less than 100 results.

## Discussion

In the present study, we aimed to investigate performance differences between athletes born in various quartiles of the year in each competition class in sprint (60 meter) and middle-distance running (600 meter), for both female and male track and field athletes. Moreover, we wanted to explore the OR of being among the top-100 athletes in each of the competition class for those born in first vs. last quartile of the year. Our main findings were that being born in the first quartile was an advantage for both sprint and middle-distance running success, especially in the youngest age classes. The RAE was present in more of the competition classes in sprint versus middle-distance running, and in more competition classes in males than in females.

This is to the best of our knowledge, the first study investigating result performance differences among athletes born in different quartiles of the year in all competition classes from 10 years to senior for both sexes. Our results indicate that chronologically older children and adolescents of both sexes have an advantage in youth track and field both in sprint and middle-distance running. Better performance in both sprint and middle-distance running among athletes born early in the year compared to those born late can probably be explained by the fact that the chronologically oldest athletes in each competition class, on average, are more biologically mature and therefore have more developed anthropometric and physical attributes that aid performance [5–9, 29–32]. Indeed, another possible explanation for our findings could also be linked with possible skewed birth date distribution in the greater Norwegian population. Thus, we checked the Medical Birth Registry of Norway (MBRN) (http://statistikkbank.fhi.no/mfr/) for quarterly distribution of births in Norway between 1996 and 2010, and found that 24.8%, 26.0%, 26.1% and 23.0% were born in Q1-Q4 respectively. Even though the number of births was not totally equal between quartiles, it is highly unlikely that the RAE in the present study can be explained by the distribution of births. Our findings are thus probably linked to the maturational hypothesis.

In sprint, the advantage of being born early in the year lasted longer in males than in females. Better results for those born early in the year compared to those born late was seen until competition class 16 years in females, and until competition class U20 in males. In boys, higher OR of being among the top-100 sprinters when born in the first quartile compared to the last quartile was seen in most competition classes and even at senior level. Highest OR among males was found in the competition class 14 years, where it was 11 times more likely to be among the top-100 athletes for those born in the first quartile of the year compared to those born in the last. In girls, higher OR was only seen in the competition classes 10, 11 and 14 years, with the highest OR in the youngest competition class. The above-mentioned sex differences may be explained by the timing of puberty. The timing of puberty could be one explanation for the high OR for boys in the age of 12 to 14 years in explosive events like 60m sprint, and also an explanation for high OR in earlier ages (10 and 11 years) in girls. Therefore, we speculate that the competition classes with high OR are partly due to the fact that these age groups have larger differences in physical capacity between athletes that are born early and late in the year. Indeed, girls enter puberty at a younger age compared to boys [33], and the longer occurrence and larger RAE in males may be explained by the later onset of puberty and the more pronounced increase in muscle mass which is an advantage in explosive events [34, 35]. Further, increased motivation, more systematic training and better training facilities as a consequence of success in adolescence may explain the persistence of RAE in the present study after puberty among male senior sprinters [11, 13–15].

Kearney and colleagues (2018) found a similar trend among males at highest performance level in sprint in the UK, showing higher OR of being born in the first quartile of the year compared to the last in all competition classes (U13, U15, U17, U20 and senior), with the highest OR in U15 (13 and 14 years old). Also, Romann and colleagues (2015) found higher OR among Swiss boys between 8 and 15 years at highest performance level in sprint. Among girls, Kearney and colleagues (2018) showed higher OR in the competition classes U13, U15 and U17, with the highest OR among the youngest (11 and 12 years old). Unlike the present study, they found RAE in girls over 14 years of age. The longer persisting RAE among girls in the study by Kearney and colleagues (2018) may be due to the different organization of competition classes in the UK with two year-bands instead of annual competition classes as in Norway.

In middle-distance running, significant better result among athletes born in the first quartile of the year compared to those born in last quartile was seen until competition class 14 years in males. In middle-distance running, significant better result among athletes born in the first quartile of the year compared to those born in last quartile was seen until competition class 14 years in males. In girls RAE was present up to the competition class 16 years, with exception of in competition the classes 14 and 15 years. The OR of being among the top-100 middle distance runners when born in first quartile compared to last quartile was in the present study higher in most competition classes until the age of 14 years in boys. Highest OR was seen in the competition class 13 years, where it was 5 times more likely to be among the top-100 athletes for those born in the first quartile of the year compared to those born in the last. Kearney and colleagues (2018) found higher OR in male middle distance running in the competition classes U13, U15 and U17 in the UK. Highest OR was found in the competition class U15 (13 and 14 years old), which is in line with our findings. However, again we cannot exclude that the different organization of competition classes between Norway and the UK can explain the longer existence of RAE in the UK. In girls, OR progressively decreased from the competition class from 10 to 13 years. Similar, Kearny and colleagues (2018) found higher OR in girls U13 (11 and 12 years old) and U15 (13 and 14 years old), with the highest OR among the youngest. As in sprint highest OR was seen at earlier age in girls than in boys, and may be due to earlier puberty onset in girls as mentioned above [33]. Unlike in male sprint, RAE did not last into adulthood in middle-distance running.

The advantage of being born early in the year lasted longer in sprint than in middle-distance running in males, indicating that puberty affects performance in sprint and middle-distance running differently in males. This is most likely due to the different physical demands in the two events. In sprint, force and power are important factors for performance [29, 32], whereas maximal oxygen uptake is important in middle-distance running [36]. The increase in body mass/muscle mass during puberty improves force and power relevant for sprint events. Although the increase in muscle mass associated with growth and maturation facilitates the use of oxygen and thus improves the absolute VO_2max_ (L·min^-1^), the concomitant increase in body mass results in an almost unchanged VO_2max_ with age during puberty when expressed in relation to body mass (ml·kg^-1^·min^-1^) [37, 38]. For girls, increased body mass during puberty is not necessarily an advantage in explosive events or in aerobic events as more body fat mass is accumulated compared to boys [38]. Increased body mass may affect the occurrence of RAE, as those born early in the year, on average, might gain weight before those born later in the year if entering puberty earlier. One causal explanation for the decreasing OR at older age in this study may be because the relative age difference in each age group are lower at higher age. i.e. the relative age difference within an age group are higher for the 10-year-old athletes, than for athletes that are twenty years old.

The stronger RAE found, especially in males, in explosive events compared to in endurance events, have previously been shown by others [23, 39]. Stronger RAEs among males at higher level athletes than in females as in the present study are also in accordance with previous studies [18, 20–23, 39]. However, some researchers have proposed strategies to limit the RAE in track and field. For instance, Romann and colleagues (2015) showed a very small or no RAE in sprint among 8 to 15 years old boys when correcting for relative age within each chronological age group, so that children were divided into age groups based on rotating cut-off dates. More recently, Brustio and Boccia [24] investigated an approach where they applied a corrective adjustment procedure in 16- and 17-year-old male and female top-level sprinters. From their retrospective analysis based on longitudinal data, the estimated expected performance changes for each annual group minimized or removed the RAE. Specifically, the results suggested an annual percentage performance difference between males of 2.02% - 2.23% and for female of 1.65% - 1.78%, and that when race results were adjusted based on these findings a more equal birth date distribution were evident compared to uncorrected performance times. Thus, to avoid that child born late in the year always are the youngest in their competition class with lesser chance to succeed, it could be possible to either adjust their performance times based on previous longitudinal data [24] or implement an alternative competition structure where competition is based on rolling cut-off birth dates so that children alternate in being the oldest and youngest in their age group [18]. These strategies may reduce drop-out in athletes born late in the year.

In conclusion, being born early in the year in events with high demand for specific physical capacities is an advantage in both sexes in most of the youngest competition classes. The age with the highest odds of being among the top-100 athletes corresponds with the age where growth and maturation naturally affect the ability to perform physically in both sexes. Although RAE and maturation are two different constructs, our findings propose that the older individuals in each competition class might have benefitted from natural improvements in performance due to puberty. It is important that both athletes, parents and coaches are aware of the RAE, and focus on mastery and progress in training and competitions regardless of performance level. Identifying talents at an early age is a difficult task thus the main goal for practitioners in track and field events dependent on high physical capacities should be to ensure that all athletes are given a chance to reach their potential, regardless of chronological age or maturational status. This could keep children and adolescence within the sport for longer, which also has implications for lifelong physical activity. It will be of interest in a further study to investigate the occurrence of RAEs in events as hurdle, high jump and shot-put where technique may be of greater importance than physical capacities.

## Supporting information

S1 Table Number of athletes born in each quartile who ran 60m. (TIF)

S2 Table Number of athletes born in each quartile who ran 600m. (TIF)
