# Supplementary figures and images for: Performance in youth track and field is associated with birth quartile. A register-based study among athletes in Norway from 10 years to senior level
Source: PLoS One. 2022 Sep 6;17(9):e0273472. doi: 10.1371/journal.pone.0273472 (PMC9447911; doi:10.1371/journal.pone.0273472)

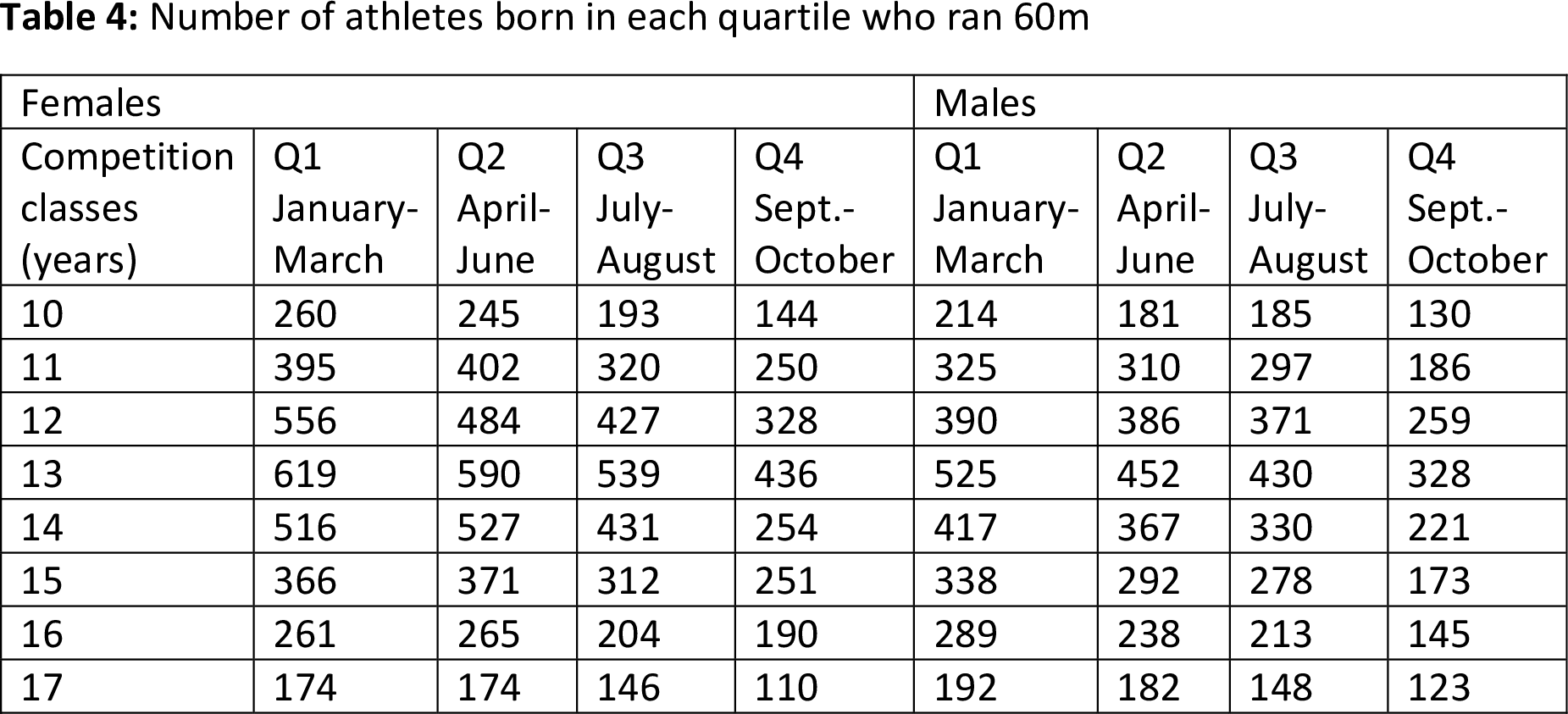

Supplement: S1 Table — (TIF) [file pone.0273472.s001.tif]

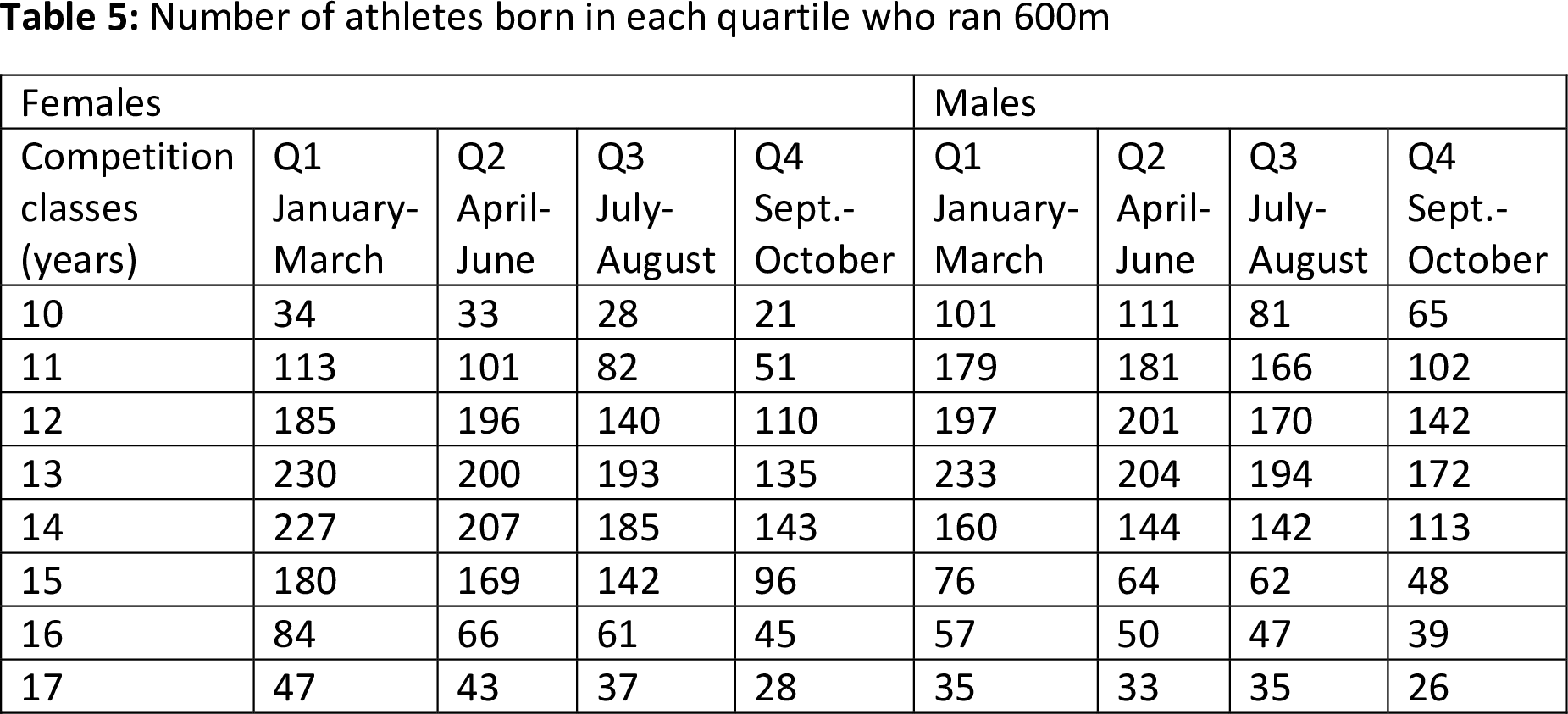

Supplement: S2 Table — (TIF) [file pone.0273472.s002.tif]
